# Gender Differences in Psychological Well-Being and Health Problems among European Health Professionals: Analysis of Psychological Basic Needs and Job Satisfaction

**DOI:** 10.3390/ijerph15071474

**Published:** 2018-07-12

**Authors:** Diego Gómez-Baya, Ana M. Lucia-Casademunt, José A. Salinas-Pérez

**Affiliations:** 1 Department of Social, Developmental and Educational Psychology, Universidad de Huelva, 21007 Huelva, Spain; diego.gomez@dpee.uhu.es; 2 Department of Business Management, Universidad Loyola Andalucía, 14004 Córdoba, Spain; 3 Department of Quantitative Methods, Universidad Loyola Andalucía, 41014 Sevilla, Spain; jsalinas@uloyola.es

**Keywords:** health professionals, job satisfaction, well-being, physical health, self-determination theory

## Abstract

*Background*: The aim was to examine the mediating role of basic psychological needs and job satisfaction in the relationship between the gender effect on health problems and psychological well-being for health professionals in Europe in 2015. *Methods*: Two multiple partial mediation analyses were conducted in order to test the partial mediation of both basic needs and job satisfaction, with gender as the independent variable and health problems or well-being, respectively, as the dependent variables, with a sample of health professionals. *Results*: Women reported lower psychological well-being and more health problems than men. The total effect of gender on both well-being and health problems was found to be significant. Regarding multiple mediation analyses: (a) the effect of gender on well-being was fully mediated by global basic need satisfaction and job satisfaction, such that gender did not present a significant direct effect and (b) the effect of gender on health problems was partially mediated by global basic need satisfaction and job satisfaction, such that the direct effect remained significant. *Conclusions*: The fulfillment of basic needs for autonomy, competence, and relatedness, as postulated within self-determination theory, was hypothesized to play a mediating role in the relationship between gender and well-being. Since significant gender differences in basic need satisfaction were observed, such a mediator should be controlled in order to achieve a significant relationship between gender and well-being when basic needs comes into play. The current study adds to the research emphasizing the need for satisfaction as a promising mechanism underlying for female health professionals’ well-being.

## 1. Introduction

Research on physical and mental health has consistently concluded that health professionals are an occupational group that is at risk because of the effects of psychosocial hazards or psychological difficulties, such as stress, fatigue, or burnout [1]. It is due to demographic factors, such as sex, age, or home-work interface, as well as organizational factors, such as workload, job satisfaction, job insecurity, or lack of resources [2]. Moreover, healthcare professionals suffer high rates of occupational-related distress and higher rates of suicide [3]. Indeed, an European study estimated that 12% of male and around 14% of female university hospital physicians from a sample in Sweden and Italy had suicidal thoughts due to degrading work experiences, role conflicts, and working conditions [4,5].

The basic reason could be that health professionals traditionally face high workload, time pressure, shift work, emotionally demanding tasks, or stressful environments in direct contact with patients, sometimes witnessing acute traumas and very painful situations [6].

Furthermore, previous empirical studies evidence the job dissatisfaction among professionals in health-care occupations [7,8,9]. However, job satisfaction for health professionals improves their motivation and performance and it is a vital resource for delivering efficient health service and patient satisfaction [7]. 

Numerous studies conclude that human resource (HR) management is critical in providing and ensuring high quality in the health care system [10]. Along these lines, the extent to which employees are satisfied within the immediate work environment determines the level of employee well-being [11,12,13]. Organizational research has revealed that need satisfaction is positively linked to outcomes, such as well-being in the workplace [13,14,15], and is negatively linked to health problems, such as distress [16,17] argued that well-being and ill-being are distinct but complementary, so complete well-being would be characterized by the presence of flourishing and the absence of languishing (that is, high well-being and low ill-being). The present research follows this approach by examining characteristics that contribute to the presence of positive psychological well-being and the absence of health problems.

Therefore, the difficult work environments of health professionals should be addressed through HR practices that focus on finding and retaining efficient and effective professionals to empowering health professionals [18], and on manage them as humans, all of which is particularly relevant for meeting basic needs in line with self-determination theory (SDT) [19]. SDT is a useful conceptual tool that complements traditional work motivation theories in order to better understand the determinants of employee outcomes in the workplace and the positive impact of certain HR practices [20]. The validity of SDT has been observed in multiple domains in which researchers have analysed people’s psychological needs as fundamentals of personal growth and integration, optimal functioning, well-being, and social development [19]. SDT has found important support in its application to the workplace environment [21,22]. In the workplace, the literature has shown positive links between needs satisfaction, well-being, and physical health [23]. In the workplace, the literature has shown positive links between needs satisfaction, well-being, and physical health [24]. In short, if employees are intrinsically motivated because they experience psychological need satisfaction within their work context, subsequent positive consequences for their psychological well-being are developed. Some authors argued that there are universal human needs and that their fulfilment is likely to enhance a person’s feelings of well-being [25]. Based on a review of the literature on SDT [26], the most important needs for individuals’ optimal growth and development are autonomy, relatedness, and competence to predict motivation and adjustment in the workplace in terms of well-being and performance [15]. In addition, the most effective HR practices should address such basic psychological needs of employees. The need for autonomy reflects the need for individuals to take action on their own behalf by making their own choices [23,27]. Competence is the extent to which individuals carry out a task to the best of their ability and achieve the desired outcomes in their environment [27]. Finally, the need for relatedness concerns the degree to which individuals feel connectedness, acceptance, and relatedness with others [28]. In line with this, some studies posit that autonomy, competence, and relatedness are individually positively related to employees’ optimal functioning [29]. Competence needs can be satisfied through HRP, such as selective hiring and extensive training, since they provide employees with knowledge of all relevant aspects of the organization. Moreover, according to Sheldon et al. [20], flat hierarchies and teamwork should boost employees’ relatedness with other employees in the organization. In addition, Sheldon et al. [20], also highlight the importance of self-managed teams, job participation, decentralized decision-making, and horizontal and vertical communication of relevant information about the organization in order to ensure feelings of autonomy among employees. Taken together, then, this set of HR practices should provide a context that allows for health professionals to fulfill their psychological needs contributing to greater job satisfaction [20].

To our knowledge we are not aware of any study guided by SDT theory that has analysed the mediating role of basic psychological needs satisfaction and job satisfaction with work conditions in the relationship between gender and health and well-being in the health professional context from different European countries. Therefore, further research is necessary to uncover whether this association function in the same way between the two sexes in a predominantly female sector. In this sense, Javadi et al. [30] concluded that women make up approximately 75% of the health workforce, which might be accompanied by differences in working patterns. For example, Reamy and Pong [31] reported that female physicians would need more flexible work schedules than their male counterparts or even to develop different styles of health care provision that require different levels of patient participation.

This study proposes drawing on SDT in satisfying the basic psychological needs of competence, relatedness, and autonomy strongly contributes to predicting health professionals’ job satisfaction, physical health, and well-being. Therefore, this study aimed to examine the mediating role of basic psychological needs and satisfaction with work conditions in the relation between the gender effect on health problems and psychological well-being for health professionals in European countries in 2015. For this purpose, first, gender differences in psychological distress and health problems in health professionals were analysed by *t*-test in order to determine whether they are significant in different European countries. Second, two multiple partial mediation analyses were conducted to test the partial mediation of both basic needs’ satisfaction and satisfaction with job conditions, with gender as the independent variable and health problems or psychological well-being, respectively, as the dependent variables. Based on the conceptualization above, the current study hypothesized that women migh thave lower levels of all three forms of need satisfaction and job satisfaction, which would in turn predict lower psychological well-being and more health problems. 

## 2. Methods

### 2.1. Study Sample

Data for the present research were collected from the 6th European Working Conditions Survey (EWCS) administered in 2015 by the European Foundation for the Improvement of Living and Working Conditions. The survey provides insight into the working environment and employment situation throughout 35 countries. The target population was all residents of the countries that are mentioned above aged 15 or older (16 or older in Bulgaria, Norway, Spain, and the UK) and was employed at the time of the survey. In light of the objective of this investigation, we obtained a subsample of 1774 health professionals (International Standard Classification of Occupations-ISCO Code 22). All of these groups are included in the Statistical Classification of Economic Activities in the European Community (commonly referred to as NACE), NACE Code 85: Health and social work. Data are available through Eurofound website [32].

The subsample consists of 1466 women and 307 men (one missing value), who are, on average, 43.71 years of age. With regard to their educational level, 473 individuals had accomplished at least secondary education level (26.7%), 157 had reached post-secondary non-tertiary education (8.9%), and 1143 respondents had completed tertiary education (64.4%). The employees’ current job tenure was approximately 13.6 years, 58.5% of jobs were in the public sector, and the largest percentage of employees was concentrated in large organizations with more than 250 employees (45.6%).

### 2.2. Measurement

Table 1 shows the EWCS questions about basic psychological needs (autonomy, competence, and relatedness) and outcome variables (job satisfaction and health problems), as well as how they are measured. The assessment of basic psychological were needs was based on the items selected in a recent work by Gomez-Baya and Lucia-Casademunt [33]. These authors demonstrated the factorial validity and notable reliability of this measure to examine the basic psychological needs of autonomy, competence, and relatedness in European employees. Mean scores were calculated for each scale of basic psychological needs and in the scale of psychological well-being, while scores for health problems were calculated by adding the responses for all items. Moreover, an average overall score was calculated for basic psychological need satisfaction from the scores for autonomy, competence, and relatedness, which will be used for partial mediation analyses.

### 2.3. Statistical Analysis

Descriptive statistics and internal consistency reliability in the study variables. A maximum likelihood imputation procedure that was based on the expectation-maximization algorithm was conducted to address missing values. Correlational analyses were performed to examine associations between variables in the full sample. Pearson correlations were calculated with the scores for the scales, while the associations with the categorical measure of job satisfaction were calculated using Kendall’s tau correlations. Concerning the first aim of the study, gender differences in health problems and psychological well-being were examined by performing Student’s T-tests with the overall sample and by country. Mean differences are reported (m.d.). Gender differences were also analyzed for psychological basic needs (Student’s *t*-tests) and job satisfaction (χ^2^ test). These analyses were conducted with SPSS version 21.0 (IBM Corp, New York, NY, USA, 2012). To determine the geographical distribution of gender differences for these two variables and their significance levels throughout Europe, the results were depicted in two grayscale choropleth maps by using a Geographic Information System (GIS).

**Figure 1:**
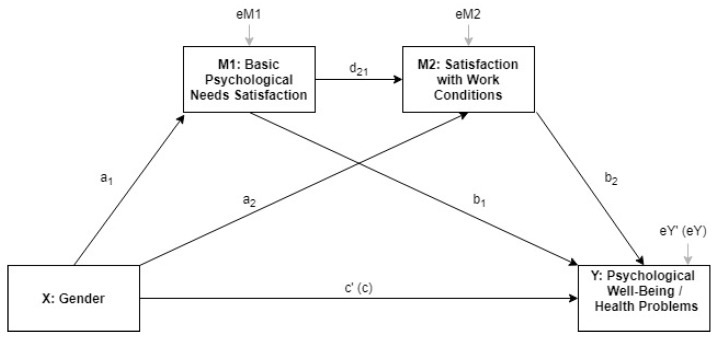
Multiple partial mediation model implemented to examine mediators in gender differences in psychological well-being and health problems.

Concerning the second aim of the study, two multiple partial mediation analyses were conducted in order to test the partial mediation of both basic needs satisfaction and satisfaction with job conditions in the gender effect on health problems and psychological well-being, respectively. In these analyses, the average overall score for basic psychological needswas introduced to simplify the analyses. A regression-based macro called Process v3.0, as developed by Hayes [34], was used following the indications that were provided by Preacher and Kelley [35]. Specifically, Model 6 was implemented (see Figure 1), in which a total of 1000 bootstrap samples were estimated for bias-corrected bootstrap confidence intervals for specific indirect effects. In this multiple partial mediation, gender was proposed as the independent variable (X), health problems or psychological well-being were presented as dependent variables (Y), and basic psychological need satisfaction (M1) and satisfaction with work conditions (M2) were introduced as partial mediators. The direct effects of gender (c’), basic psychological needs’ satisfaction (b_1_), and satisfaction with work conditions (b_2_), on Y (i.e., health problems or psychological well-being) were analysed. Furthermore, the total effect of gender on Y was calculated (c), as well as gender effects on basic psychological need satisfaction (a_1_) and on satisfaction with work conditions (a_2_). The effect of basic psychological needs’ satisfaction on satisfaction with work conditions was also examined (d_21_). These regression analyses were carried out with the full sample of European health professionals and by country, with F statistics and R^2^ values reported and statistical significance at *p* < 0.05. Moreover, countries were grouped according to welfare state regimes, following previous studies [36,37]. The results of these regression analyses were presented for conservative regimes (i.e., Austria, Belgium, France, Germany, Luxembourg, Netherlands, and Switzerland), post-communist regimes (i.e., Bulgaria, Croatia, Czech Republic, Estonia, Hungary, Latvia, Lithuania, Poland, Romania, Slovakia, Slovenia, Montenegro, FYROM, Serbia, and Albania), Mediterranean regimes (i.e., Cyprus, Greece, Italy, Malta, Portugal, Spain, and Turkey), social-democratic regimes (i.e., Denmark, Finland, Sweden, and Norway), and liberal regimes (i.e., Ireland and UK).

## 3. Results

### 3.1. Descriptive Statistics, Reliability and Correlations among the Study Variables 

Table 2 presents the descriptive statistics (minimum, maximum, mean, and standard deviation) for the study variables, as well as internal consistency reliability and correlations. Correlation analyses indicated that psychological need satisfaction was positively associated with satisfaction with working conditions and psychological well-being and negatively related to health problems. Moreover, satisfaction with working conditions presented a positive correlation with psychological well-being and a negative correlation with health problems. Regarding reliability, acceptable values for Cronbach’s alpha were found for basic psychological needs and health problems, and a significant result was observed for the assessment of psychological well-being. 

Table 2 also reports the results of the analyses of gender differences in the study variables in the full sample. In the full sample of European health professionals, women reported lower psychological well-being (M = 4.42, SD = 0.99) than men (M = 4.63, SD = 0.90). Moreover, women presented more health problems (M = 2.60, SD = 2.09) than men (M = 1.90, SD = 1.96). Concerning basic psychological needs, women reported lower scores in autonomy (M = 3.06, SD = 0.95) and competence (M = 4.34, SD = 0.53) than men (M = 3.46, SD = 0.95, and M = 4.43, SD = 0.53, respectively), but no difference in relatedness was found. When gender differences in the overall score for basic need satisfaction were examined, the results indicated that women presented lower score (M = 3.80, SD = 0.55) than men (M = 3.93, SD = 0.54). In terms of satisfaction with work conditions, 14.3% of women reported that they did not feel very satisfied, while this percentage was 9.8% in men. Moreover, only 27.4% of women indicated that they were very satisfied with their working conditions, and this percentage was 34.3% in the subsample of men.

**Figure 2:**
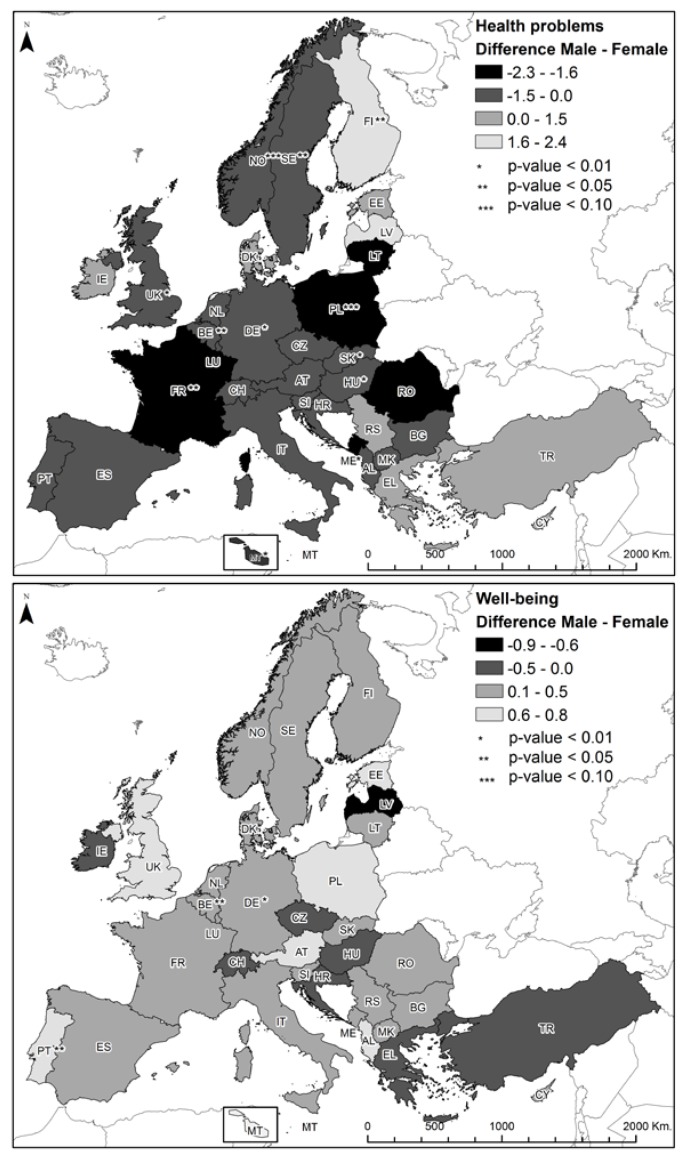
Maps of gender differences in health problems and psychological well-being among European health professionals. Source: Own elaboration from the data of the 6th EWCS 2016. © European Foundation for the Improvement of Living and Working Conditions (Eurofound), 2018 [41].

The means and standard deviations of the study variables and the sample composition by country and by welfare state regime are described in Table A1. Figure 2 shows the gender difference in psychological well-being and health problems in each participating European country, indicating the significance and size of the mean differences. Greater gender differences in health problems were observed in Poland, Montenegro, and France. The differences were also significant in Malta, United Kingdom, Slovakia, Norway, Germany, Hungary, Belgium, and Sweden. However, more health problems were reported by men in Finland. Regarding psychological well-being, significant gender differences were observed in Portugal, Belgium, and Germany, with women showing lower scores than men. It is also noteworthy that the differences in the two variables are significantly unfavourable for female professionals in Germany and Belgium. Concerning welfare state regime, women presented lower psychological well-being in conservative regimes, *t*(535) = 3.00, *p* = 0.003, *md* = 0.30. More health problems were reported by women in conservative regimes, *t*(535) = −3.79, *p* < 0.001, *md* = −0.90, and in post-communist regimes, *t*(499) = −3.57, *p* < 0.001, *md* = −0.93.

### 3.2. Multiple Partial Mediation of Basic Needs Satisfaction and Satisfaction with Job Conditions in the Gender Effect on Psychological Well-Being

**Figure 3:**
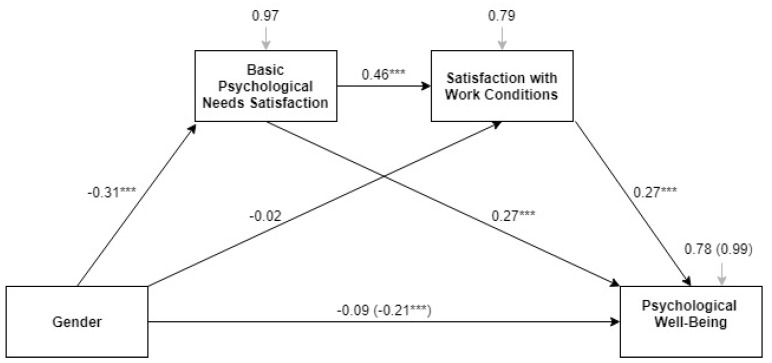
Model of the multiple mediation of basic needs and satisfaction with work conditions in the effect of gender on psychological well-being among European health professionals. Note: *** *p* < 0.001. Source: Own elaboration from the data of the 6th EWCS 2016. © European Foundation for the Improvement of Living and Working Conditions (Eurofound), 2018 [41].

Figure 3 describes the model of multiple mediation from the overall score for basic needs satisfaction and satisfaction with work conditions in the examined effect of gender on psychological well-being in the full sample of European health professionals (β and *p* values are indicated). First, the total effect of gender (represented between parentheses) on psychological well-being (*t* = −3.66, se = 0.06, LLCI = −0.33, ULCI = −0.10) was found to be significant, F(1, 1771) = 13.40, *p* < 0.001, R^2^ = 0.01. Second, the relationships between gender and psychological well-being were found to be fully mediated (*t* = −1.66, se = 0.05, LLCI = −0.19, ULCI = 0.02) by the direct and positive effects of need satisfaction (t = 10.52, se = 0.03, LLCI = 0.22, ULCI = 0.33) and the satisfaction with work conditions (*t* = 10.53, se = 0.03, LLCI = 0.22, ULCI = 0.32), F(3, 1769) = 137.60, *p* < 0.001, R^2^ = 0.22. Furthermore, there were significant effects of gender on need satisfaction (*t* = −5.07, se = 0.06, LLCI = −0.43, ULCI = −0.19), F(1, 1771) = 25.67, *p* < 0.001, R^2^ = 0.01, and by need satisfaction on satisfaction with work conditions (*t* = 19.99, se = 0.02, LLCI = 0.42, ULCI = 0.51), F(2, 1770) = 205.88, *p* < 0.001, R^2^ = 0.21. Thus, lower scores for need satisfaction were observed in women (M = 3.80, SD = 0.54) than in men (M = 3.93, SD = 0.55), *t*(1441) = 3.37, *p* = 0.001, m.d. = 0.13, and need satisfaction had a positive impact on the satisfaction with work conditions. No significant gender differences in satisfaction with work conditions were observed (*t* = −0.33, se = 0.06, LLCI = −0.13, ULCI = 0.09). Table 3 presents the results of the direct effect model in order to explain psychological well-being by country and by welfare state regime, while Table A2 describes the results of the total effect model and the effects of the mediators by country and by welfare state regime. Need satisfaction and satisfaction with work conditions were found to fully mediate the effect by gender on psychological well-being in all welfare state regimes. 

### 3.3. Multiple Partial Mediation of Basic Needs Satisfaction and Satisfaction with Job Conditions in the Gender Effect on Health Problems

**Figure 4:**
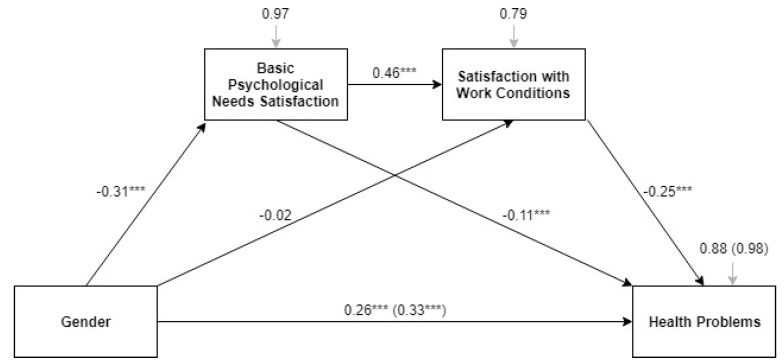
Model of the multiple partial mediation of basic needs and satisfaction with work conditions in the effect of gender on health problems among European health professionals. Note: *** *p* < 0.001. Source: Own elaboration from the data of the 6th EWCS 2016. © European Foundation for the Improvement of Living and Working Conditions (Eurofound), 2018 [41].

Figure 4 presents the model for multiple mediation by the overall score for basic need satisfaction and satisfaction with work conditions in the relationship between gender and health problems, with β and *p* values indicated. First, the total effect of gender on health problems (between parentheses) was significant (*t* = 5.60, se = 0.06, LLCI = 0.21, ULCI = 0.45), F (1, 1771) = 31.31, *p* < 0.001, R^2^ = 0.02. Second, the three direct effects on health problems were also significant, F(3, 1769) = 71.71, *p* < 0.001, R^2^ = 0.11. Thus, the gender effect remained significant (*t* = 4.54, se = 0.06, LLCI = 0.15, ULCI = 0.37), when the mediation of basic needs (*t* = −3.94, se = 0.03, LLCI = −0.16, ULCI = −0.05) and satisfaction with work conditions (*t* = −9–39, se = 0.03, LLCI = −0.30, ULCI = −0.20) were included in the model. The negative effects of both mediators were found to partially mediate the gender differences in health problems in the full sample. Thus, lower need satisfaction and lower satisfaction with work conditions explained more health problems. The effects on the mediators are similar to those that are described in the previous section. The direct effect models and the total effect models to explain health problems by country and by welfare state regime are shown in Table 3 and Table A2, respectively. Gender effect on health problems was significant after the inclusion of the mediators in conservative and post-communist regimes, while it was not significant in social-democratic, Mediterranean, and liberal regimes. The role of need satisfaction on health problems was especially important in social-democratic, Mediterranean, and liberal regimes.

## 4. Discussion

This study seeks to contribute to the literature by exploring the mediating role of basic psychological needs and job satisfaction in the relation between the gender effect on health problems and psychological well-being in the health professional work context in Europe. In the present study, the levels of psychological well-being, health problems, and basic psychological need satisfactions employees in the European health sector differed between genders, whereas the levels of job satisfaction did not. The significant findings are that basic psychological needs satisfaction partially mediated the relationships between the gender effect and health problems and fully mediated the relationships between gender and psychological well-being. This result suggests that basic need satisfaction might play a more central role in exclusively mediating the link between gender and psychological well-being. As shown in Figure 1, when the indirect paths were tested, gender and psychological well-being were significantly positively linked through basic need satisfaction. In this sense, the current study uncovered links between gender effects and psychological well-being consistent with the central role that these three psychological needs play in promoting individual well-being [42]. Thus, female health professionals who feel more autonomous, more able and competent, and more connected to the people in their professional lives would report higher levels of psychological well-being. This result implies that if basic psychological needs were satisfied, women health professionals could still benefit from higher levels of psychological well-being in the workplace. The mediation of need satisfaction and satisfaction with work conditions in the effect by gender on psychological well-being was consistent in the different welfare state regimes. Regarding health problems, gender differences were observed in conservative and post-communist regimes, with no effect being observed by need satisfaction. However, in social-democratic, Mediterranean, and liberal regimes need satisfaction showed a negative effect on health problems and fully mediated the effect by gender. The health differences among European countries by welfare state regimes were also observed in previous studies, such as Eikemo et al. [36] and Alvarez-Galvez [37]. Contrary to this finding are the conclusions by Deci et al. [23], who indicated that the employees’ experiences, which satisfied the needs for autonomy, competence, and relatedness correlated with improved well-being at work similarly across gender groups. However, the issue of gender differences in basic need satisfaction and psychological well-being has not received much empirical attention. 

On the other hand, this finding is consistent with previous studies that provide scientific evidence that health professionals are an occupational group at risk for psychological impairments and job dissatisfaction because of their high work [43]. Indeed, there is increasing evidence that health professionals experience high levels of stress and psychological distress [44,45]. Occupational distress has been studied mainly for physicians [46,47,48] and nurses [49,50]. However, literature has found differences in other professional categories [51], as well as between healthcare settings [52]. Along these lines, different factors have been suggested to explain the difficulties that are encountered by health professionals. Although most of these factors are stressors, such as heavy workload, patient management, financial concerns, negative family-work spillover, exposure to patients’ suffering and death, the possibility of making professional mistakes, and the threat of lawsuits [53], researchers also have considered demographic factors, such as gender [2,46,54]. Our findings may explain the previous research finding that gender discrimination has a more negative impact on the well-being of women at work [55]. Research on gender differences in psychological well-being suggests that female health professionals tend to experience lower job satisfaction and subjective well-being than men [53], as well as higher rates of suicide than their male colleagues [4,53].

Future studies should continue searching for other potential mediators that may contribute to the relationship between gender and psychological well-being and health problems in the organizational and workplace contexts. 

From a practical perspective, our research based on SDT [12] focused on basic psychological need satisfaction (i.e., autonomy, relatedness, and competence) and job satisfaction as the key mechanisms that are accounting for the relationship between gender and psychological well-being and health. In this sense, we recommend creating environments that promote psychological well-being and health for optimal functioning in the work context, aligned with individual differences among employees. Therefore, the study results suggest the need to introduce organizational interventions that are based on positive organizational psychology to satisfy basic needs—an approach that is based on promoting strengths to improve health and well-being. These interventions must take into account the possible barriers, constraints and challenges that women face in the workplace, specifically in the health professional sector, in the satisfaction of their basic psychological needs. Barriers result from gender differences related to salary gaps, glass ceilings, work-life balance, aggression, or harassment. Thus, if gender differences in psychological well-being arise from gender differences in basic need satisfaction, promoting better care to satisfy basic psychological needs for women in the health professional sector could contribute to eliminating those differences in their psychological well-being.

## 5. Conclusions

- Female health professionals in Europe reported lower psychological well-being, lower levels of basic psychological need satisfaction, and more health problems.
- Basic psychological need satisfaction is an underlying mechanism for explaining gender differences in psychological well-being.
- Basic psychological need satisfaction partly explains gender differences in health problems.

These results support the application of Self Determination Theory (SDT) in the workplace health context and confirm its value in contributing strongly to predict health professionals; job satisfaction, physical health, and well-being.

## Figures and Tables

**Table 1:** Items related to basic psychosocial needs and outcomes in the 6th European Working Conditions Survey (EWCS) 2016.

Instruments to Assess Variables	Basic Psychological Needs			Outcome Variables		
	Autonomy	Competence	Relatedness	Job Satisfaction ^1^	Health Problems	Psychological Well-Being Well-Being Index (WHO-5) ^2^
Items from 6th EWCS	You can influence decisions that are important for your work?	Your job gives you the feeling of work well done?	You are treated fairly at your workplace?	On the whole, are you very satisfied, satisfied, not very satisfied or not at all satisfied with working conditions in your main paid job?	Over the last 12 months, did you suffer from any of health problems? Hearing problems, skin problems, backache, muscular pains in the shoulders, neck and/or upper limbs, muscular pains in lower limbs, headaches or eyestrain, injury/ies, anxiety, overall fatigue, and other.	How you have been feeling over the last two weeks. I have felt cheerful and in good spirits
	You are involved in improving the work organisation or work processes of your department or organisation?	You have the feeling of doing useful work?	Your colleagues help and support you?			I have felt calm and relaxed
	You are consulted before objectives are set for your work?	You know what is expected of you at work?	Your manager helps and supports you?			I have felt active and vigorous
	You are able to apply your own ideas in your work?	You have enough time to get the job done?				I woke up feeling fresh and rested
	You have a say in the choice of your work colleagues?					My daily life has been filled with things that interest me
Likert-Type	Valued from 1 to 5: never, rarely, sometimes, most of the time, and always.			Valued from 1 to 4: not all satisfied, not very satisfied, satisfied, and very satisfied	Two response options are offered (yes/no)	Valued from 1 to 6: at no time, some of the time, less than a half of the time, more than a half of the time, most of the time, and all of the time.

^1^ As some authors argue, validity exists for single-item measures, and their findings provide qualified support for them [38,39]. Accordingly, empirical literature ratifies the use of a single variable when studying job satisfaction. ^2^ Index based on the eudemonic approach. WHO-5 well-being index have been validated in previous research [40]. Source: 6th EWCS 2016. © European Foundation for the Improvement of Living and Working Conditions (Eurofound), 2018 [41].

**Table 2:** Descriptive statistics, internal consistency reliability, gender differences, and correlation analyses among study variables in the total sample of health professionals.

Variables	Min–Max	M(SD)	M(SD) Women	M(SD) Men	Gender Differences	1	2	3	4	5	6	7
1. Autonomy	1–5	3.13 (0.94)	3.06 (0.93)	3.46 (0.95)	*t*(1562) = 6.26, *p* < 0.001, *md* = 0.40	(0.78)						
2. Competence	1.25–5	4.36 (0.53)	4.34 (0.53)	4.43 (0.53)	*t*(1753) = 2.67, *p* = 0.008, *md* = 0.09	0.26 ***	(0.63)					
3. Relatedness	1–5	4.08 (0.71)	4.08 (0.72)	4.11 (0.69)	*t*(1530) = 0.59, *p* = 0.556, *md* = 0.03	0.36 ***	0.45 ***	(0.66)				
4. Basic Need Satisfaction	1.28–5	3.82 (0.54)	3.80 (0.54)	3.93 (0.55)	*t*(1441) = 3.37, *p* = 0.001, *md* = 0.13	0.79 ***	0.66 ***	0.79 ***	(0.78)			
5. Satisfaction with Work Conditions	1–4	3.12 (0.69)	3.10 (0.69)	3.21 (0.68)	χ^2^(3) = 8.23, *p* = 0.042, *V* = 0.07	0.29 ***	0.32 ***	0.41 ***	0.43 ***	1		
6. Psychological Well-being	1–6	4.46 (0.98)	4.42 (0.99)	4.63 (0.90)	*t*(1763) = 3.43, *p* = 0.001, *md* = 0.21	0.23 ***	0.36 ***	0.35 ***	0.40 ***	0.40 ***	(0.88)	
7. Health Problems	0–10	2.48 (2.09)	2.60 (2.09)	1.90 (1.96)	*t*(1752) = −5.37, *p* < 0.001, *md* = −0.70	−0.14 ***	−0.18 ***	−0.21 ***	−0.23 ***	−0.30 ***	−0.38 ***	(0.70)

Note: *** Significant at level *p* < 0.001. Source: Own elaboration from the data of the 6th EWCS 2016. © European Foundation for the Improvement of Living and Working Conditions (Eurofound), 2018 [41].

**Table 3:** Direct effect models to explain psychological well-being and health problems, on the basis of gender, basic needs, and satisfaction with work conditions, by country and by welfare state regime.

Country and Welfare State Regime	Code	Direct Effect Model: Wellbeing					Direct Effect Model: Health Problems				
		R^2^	F	c’	b_1_	b_2_	R^2^	F	c’	b_1_	b_2_
Conservative		0.28	55.07 ***	−0.14	0.23 ***	0.39 ***	0.15	31.85 ***	0.37 ***	−0.05	−0.33 ***
Austria	AT	0.32	4.51 **	−0.20	0.15	0.46 **	0.33	5.33 **	0.21	−0.24	−0.42 **
Belgium	BE	0.39	26.12 ***	−0.11	0.28 **	0.53 ***	0.18	12.93 ***	0.37	−0.09	−0.33 **
France	FR	0.26	6.90 ***	−0.14	0.42 **	0.18	0.14	4.44 **	0.59	−0.39 **	0.06
Germany	DE	0.29	15.03 ***	−0.19	0.37 ***	0.10	0.09	6.67 ***	0.55 **	0.05	−0.22 **
Luxembourg	LU	0.15	3.01 *	−0.18	0.03	0.42 *	0.21	4.18 *	0.24	−0.12	−0.44 **
Netherlands	NL	0.41	14.56 ***	−0.51 **	−0.04	0.52 ***	0.13	7.20 ***	0.35	0.01	−0.36 **
Switzerland	CH	0.19	3.96 *	0.09	0.19	0.32 **	0.20	3.43 *	0.31	0.01	−0.39 *
Post-Communist		0.17	31.94 ***	−0.10	0.28 ***	0.20 ***	0.09	20.07 ***	0.40 ***	−0.05	−0.25 ***
Bulgaria	BG	0.09	1.49	−0.03	0.05	0.28	0.46	10.14 ***	−0.02	0.42 *	−0.47 ***
Croatia	HR	0.31	5.05 **	−0.09	0.15	0.48 **	0.28	10.41 ***	0.38	0.01	−0.57 ***
Czech Rep.	CZ	0.04	1.04	0.32	0.10	0.06	0.12	1.44	0.03	0.04	−0.35 *
Estonia	EE	0.17	5.39 **	−0.74	0.50 *	−0.07	0.03	1.25	0.02	−0.15	0.20
Hungary	HU	0.25	2.29	0.24	0.30 *	0.02	0.19	4.53 **	0.67 ***	−0.05	−0.17
Latvia	LV	0.41	4.69 *	0.78	0.01	0.73 **	0.15	18.25 ***	0.05 *	0.04	−0.34
Lithuania	LT	0.31	3.86 *	0.01	0.05	0.34 *	0.13	1.99	0.51	0.01	−0.28
Poland	PL	0.14	2.69	−0.02	0.29	0.07	0.11	2.44	0.88	−0.10	−0.12
Romania	RO	0.61	13.98 ***	0.80**	0.47 ***	0.41 *	0.25	5.70 **	0.17	−0.33 *	−0.21
Slovakia	SK	0.35	4.81 **	0.64	0.83 **	0.16	0.10	3.64 *	0.47	−0.10	−0.25
Slovenia	SI	0.42	14.58 ***	−0.02	0.70 ***	0.11	0.14	6.09 **	0.57 *	−0.23	−0.11
Montenegro	ME	0.20	2.06	−0.14	−0.25	0.34 *	0.21	7.01 **	0.87 **	0.04	−0.27
FYROM	MK	0.24	4.87 **	−0.19	0.56 ***	0.11	0.15	2.78	0.51	0.22	−0.29
Serbia	RS	0.08	1.57	−0.62	0.11	0.19	0.18	4.17 *	0.23	−0.10	−0.35 *
Albania	AL	0.13	3.33 *	−0.35	0.17	0.15	0.02	0.19	0.01	0.01	−0.10
Mediterranean		0.25	26.71 ***	0.03	0.27 ***	0.30 ***	0.15	16.87 ***	0.17	−0.20 **	−0.26 ***
Cyprus	CY	0.13	2.37	−0.06	0.02	0.39 *	0.21	4.72 **	−0.04	0.12	−0.48 **
Greece	EL	0.02	0.24	0.18	0.03	−0.02	0.28	1.81	−0.37	−0.70	0.49 *
Italy	IT	0.26	5.72 **	−0.46 **	−0.11	0.43 **	0.21	7.52 ***	0.44	0.02	−0.48 ***
Malta	MT	0.57	12.75 ***	0.22	0.60 ***	0.32 **	0.25	5.75 **	0.64 **	−0.11	−0.21
Portugal	PT	0.58	9.41 **	−0.89 **	0.64 **	0.07	0.10	1.06	0.11	−0.34	−0.02
Spain	ES	0.28	14.53 ***	0.09	0.40 **	0.19	0.29	13.81 ***	0.14	−0.50 ***	−0.10
Turkey	TR	0.42	12.05 ***	0.16	0.18	0.46 **	0.19	2.20	−0.11	0.24	−0.56 *
Social-Democratic		0.22	26.46 ***	−0.13	0.39 ***	0.13	0.10	9.08 ***	0.09	−0.24 ***	−0.07
Denmark	DK	0.38	10.91 ***	−0.16	0.32 **	0.24 *	0.20	3.56 *	−0.12	−0.29 *	−0.14
Finland	FI	0.32	9.16 ***	0.05	0.18	0.38 **	0.25	4.53 **	−0.31 **	−0.37	−0.11
Sweden	SE	0.13	3.84 *	−0.06	0.35 *	0.05	0.10	3.35 *	0.28	−0.25 *	0.03
Norway	N	0.29	15.81 ***	−0.57	0.74 ***	−0.10	−13	5.17 **	0.58 **	−0.15	−0.17
Liberal		0.17	9.36 ***	−0.13	0.24 *	0.25 **	0.14	6.24 ***	0.19	−0.22 *	−0.19 *
Ireland	IE	0.19	4.35 **	0.21	0.09	0.33 *	0.10	2.47	−0.27	−0.22 *	−0.07
UK	UK	0.19	6.26 ***	−0.31	0.34 *	0.17	0.20	6.09 **	0.48 *	−0.14	−0.28 *

Notes: *** *p* < 0.001, ** *p* < 0.01, * *p* < 0.05. Source: Own elaboration from the data of the6th EWCS 2016. © European Foundation for the Improvement of Living and Working Conditions (Eurofound), 2018 [41].

## Appendix A

**Table A1:** Descriptive statistics of study variables and sample composition by country and by welfare state regime.

Countries and Welfare State Regime	Code	*n* = 1774	% Women	Needs Satisfaction		Satisfaction with Work Conditions		Psychological Well-Being		Health Problems	
				M	SD	M	SD	M	SD	M	SD
Conservative		538	82.7	3.77	0.54	3.13	0.68	4.46	1.00	2.45	2.11
Austria	AT	46	84.8	3.76	0.48	3.26	0.77	4.42	1.06	2.28	2.27
Belgium	BE	138	77.5	3.75	0.55	3.07	0.71	4.33	1.18	2.83	2.20
France	FR	73	87.7	3.74	0.43	3.00	0.61	4.27	0.87	3.26	2.17
Germany	DE	111	87.4	3.58	0.60	3.14	0.68	4.55	0.91	1.89	1.85
Luxembourg	LU	43	74.4	3.88	0.65	3.02	0.64	4.29	1.04	2.49	2.24
Netherlands	NL	72	84.7	3.98	0.42	3.18	0.66	4.78	0.74	2.40	1.95
Switzerland	CH	55	81.8	3.86	0.51	3.38	0.62	4.67	0.92	1.74	1.74
Post-Communist		511	85.7	3.90	0.56	3.06	0.67	4.43	0.95	2.53	2.06
Bulgaria	BG	23	73.9	4.10	0.45	3.26	0.69	4.30	0.97	2.57	1.38
Croatia	HR	37	81.1	3.72	0.54	2.97	0.73	4.23	0.98	2.76	2.33
Czech Rep.	CZ	39	84.6	4.09	0.54	3.44	0.55	4.73	0.79	1.62	1.64
Estonia	EE	34	97.1	4.01	0.51	3.18	0.47	4.48	1.06	3.50	1.78
Hungary	HU	43	83.7	3.92	0.78	3.00	0.85	4.75	0.81	1.16	1.44
Latvia	LV	24	95.8	3.84	0.63	3.13	0.45	4.29	0.78	2.96	1.66
Lithuania	LT	28	89.3	3.84	0.61	3.07	0.72	4.11	0.65	3.43	2.32
Poland	PL	32	90.6	3.84	0.54	3.06	0.62	4.46	0.85	3.45	2.31
Romania	RO	20	95.0	3.92	0.64	3.10	0.45	4.27	0.88	3.47	2.12
Slovakia	SK	32	90.6	3.64	0.47	3.06	0.56	4.31	1.24	2.19	2.10
Slovenia	SI	59	84.7	3.94	0.47	2.86	0.68	4.50	1.00	2.48	2.04
Montenegro	ME	33	84.8	3.81	0.55	3.13	0.66	4.44	0.88	2.21	2.03
FYROM	MK	39	76.9	4.26	0.49	3.00	0.61	4.76	1.02	2.50	1.94
Serbia	RS	34	79.4	3.75	0.53	2.81	0.82	4.04	1.07	3.23	2.15
Albania	AL	34	85.3	3.68	0.35	3.03	0.67	4.34	0.75	1.59	1.72
Mediterranean		323	74.0	3.82	0.57	3.16	0.67	4.57	0.98	2.58	2.28
Cyprus	CY	33	66.7	3.57	0.41	2.97	0.68	4.28	1.09	3.15	2.02
Greece	EL	20	70.0	3.71	0.32	3.50	0.69	4.79	0.60	2.05	2.14
Italy	IT	57	75.0	3.66	0.51	3.14	0.64	4.30	0.73	1.93	2.12
Malta	MT	53	66.0	4.00	0.50	3.11	0.70	4.28	0.96	3.48	1.77
Portugal	PT	16	81.3	4.05	0.42	3.19	0.54	4.99	0.72	0.81	1.56
Spain	ES	117	82.1	3.84	0.64	3.16	0.69	4.77	1.10	2.73	2.53
Turkey	TR	28	60.7	3.90	0.69	3.29	0.60	4.85	0.84	2.27	2.24
Social-Democratic		279	86.7	3.78	0.47	3.17	0.73	4.45	0.94	2.43	1.83
Finland	FI	39	89.7	3.88	0.46	3.18	0.76	4.53	0.89	2.85	2.23
Denmark	DK	71	87.3	3.86	0.45	3.35	0.70	4.54	0.76	2.40	1.64
Sweden	SE	87	79.3	3.67	0.50	3.02	0.75	4.39	1.00	2.17	1.79
Norway	N	82	92.7	3.77	0.46	3.16	0.69	4.39	1.05	2.51	1.81
Liberal		122	83.6	3.83	0.53	3.10	0.73	4.25	1.03	2.28	2.10
Ireland	IE	54	87.0	3.84	0.53	3.11	0.66	4.41	0.86	1.98	1.81
UK	UK	68	80.9	3.82	0.54	3.09	0.79	4.12	1.14	2.51	2.29

Source: Own elaboration from the data of the 6th EWCS 2016. © European Foundation for the Improvement of Living and Working Conditions (Eurofound), 2018 [41].

**Table A2:** Effects on mediators (i.e., needs satisfaction and satisfaction with work conditions) and total effects by gender on psychological well-being and health problems, by country and by welfare state regime.

Countries and Welfare State Regime	Code	M1: Need Satisfaction			M2: Satisfaction with Work Conditions				Total Effect Model: Wellbeing			Total Effect Model: Health Problems		
		R^2^	F	a_1_	R^2^	F	a_2_	d_21_	R^2^	F	c	R^2^	F	c
Conservative		0.05	32.84 ***	−0.58 ***	0.25	61.28 ***	0.18	0.50 ***	0.01	9.13 **	−0.31 **	0.03	17.49 ***	0.43 ***
Austria	AT	0.25	19.94 ***	−0.31 ***	0.18	2.82	0.46	0.57 *	0.03	2.21	−0.53	0.05	5.03 *	0.64 *
Belgium	BE	0.07	12.65 ***	−0.65 ***	0.35	34.07 ***	0.29	0.63 ***	0.02	3.22	−0.36	0.04	5.72 *	0.48 *
France	FR	0.04	4.41 *	−0.51 *	0.23	10.70 ***	−0.53 *	0.43 **	0.03	2.37	−0.49	0.06	4.89 *	0.75 *
Germany	DE	0.05	9.70 **	−0.74 **	0.31	13.95 ***	0.11	0.48 ***	0.03	9.81 **	−0.48 **	0.05	9.56 **	0.57 **
Luxembourg	LU	0.01	1.01	−0.32	0.16	5.05 *	0.15	0.32 **	0.01	0.25	−0.17	0.01	0.50	0.26
Netherlands	NL	0.01	0.04	−0.05	0.27	9.24 ***	0.49	0.61 ***	0.02	0.81	−0.27	0.01	0.34	0.18
Switzerland	CH	0.03	1.05	−0.41	0.24	10.73 ***	0.16	0.46 ***	0.01	0.01	0.01	0.02	2.72	0.32
Post-Communist		0.01	1.46	−0.17	0.10	27.99 ***	−0.07	0.31 ***	0.01	2.56	−0.17	0.02	15.14 ***	0.44 ***
Bulgaria	BG	0.18	6.10 *	−0.76 *	0.22	7.94 **	−0.47	0.43 *	0.02	0.56	−0.28	0.01	0.03	0.04
Croatia	HR	0.06	3.21	−0.62	0.12	2.78	0.67	0.33 *	0.01	0.01	0.04	0.01	0.05	0.11
Czech Rep.	CZ	0.04	3.15	−0.55	0.17	5.01 *	−0.23	0.31 **	0.01	0.83	0.24	0.01	0.33	0.15
Estonia	EE	0.01	1.71	0.20	0.20	67.50 ***	−0.25 ***	0.26 *	0.01	8.42 **	−0.55 **	0.01	2.77	−0.25
Hungary	HU	0.06	2.24	0.82	0.14	4.31 *	0.50	0.30 *	0.05	2.47	0.50	0.07	12.08 **	0.50 **
Latvia	LV	0.27	205.64 ***	0.76 ***	0.06	1.73	−0.25	0.16	0.06	34.36 ***	0.95 ***	0.07	42.14 ***	−0.02 ***
Lithuania	LT	0.02	0.41	0.52	0.36	13.93 ***	−0.71 ***	0.39 **	0.06	3.03	−0.48	0.07	3.34	0.95
Poland	PL	0.25	17.03	−0.66 ***	0.26	6.51 **	0.43	0.52 **	0.03	1.31	−0.53	0.09	7.59 **	0.10 **
Romania	RO	0.02	6.45 *	−0.77 *	0.23	87.50 ***	−0.35 ***	0.04	0.01	0.38	−0.12	0.03	10.26 **	0.72 **
Slovakia	SK	0.07	5.86 *	−0.74 *	0.26	5.74 **	−0.07	0.49 **	0.01	0.01	−0.04	0.04	12.06 **	0.65 **
Slovenia	SI	0.02	1.25	−0.34	0.28	9.44 ***	−0.03	0.61 ***	0.01	0.64	−0.28	0.06	7.72 **	0.68 **
Montenegro	ME	0.03	0.97	0.48	0.05	1.34	−0.56	0.13	0.03	2.74	−0.43	0.15	17.08 ***	0.02 ***
FYROM	MK	0.01	0.14	−0.14	0.02	0.27	0.02	0.15	0.01	0.82	−0.27	0.05	1.87	0.48
Serbia	RS	0.10	3.93	0.73	0.17	5.52 **	0.46	0.41 *	0.02	0.85	−0.39	0.01	0.06	−0.12
Albania	AL	0.08	3.18	−0.52	0.06	1.76	−0.56	0.14	0.06	5.74*	−0.54*	0.01	0.06	0.08
Mediterranean		0.01	2.09	−0.18	0.27	56.81 ***	0.01	0.49 ***	0.01	0.11	−0.04	0.01	3.02	0.23
Cyprus	CY	0.01	0.15	0.14	0.19	6.80 **	0.06	0.50 ***	0.01	0.01	−0.01	0.01	0.05	−0.09
Greece	EL	0.01	0.24	−0.17	0.22	2.95	−0.24	0.65 *	0.02	0.33	0.18	0.04	0.59	−0.42
Italy	IT	0.03	2.01	−0.38	0.24	7.41 **	0.28	0.48 ***	0.05	3.99	−0.38	0.03	2.52	0.39
Malta	MT	0.04	2.47	−0.40	0.24	7.10 **	0.35	0.55 ***	0.01	0.01	0.03	0.14	9.08 **	0.66 **
Portugal	PT	0.01	0.06	0.08	0.02	0.18	−0.26	0.01	0.22	9.63 **	−0.86 **	0.01	0.07	0.09
Spain	ES	0.03	5.25 *	−0.48 *	0.46	61.38 ***	−0.21	0.59 ***	0.01	0.38	−0.19	0.02	10.78	0.42
Turkey	TR	0.11	3.57	0.85	0.18	2.22	0.01	0.29	0.06	1.74	0.42	0.01	0.01	−0.03
Social-Democratic		0.01	4.01 *	−0.29 *	0.38	90.72 ***	−0.24	0.71 ***	0.01	3.61	−0.30	0.01	1.50	0.19
Denmark	DK	0.01	0.56	−0.20	0.36	20.13 ***	−0.20	0.69 ***	0.02	1.55	−0.30	0.01	0.01	−0.01
Finland	FI	0.01	0.27	−0.28	0.33	19.32 ***	−0.33	0.70 ***	0.01	0.10	−0.19	0.11	3.31	−0.15
Sweden	SE	0.07	8.56 **	−0.62 **	0.30	22.68 ***	−0.31	0.58 ***	0.02	1.53	−0.31	0.04	5.42 *	0.41 *
Norway	N	0.01	0.19	0.13	0.51	30.28 ***	−0.38	0.84 ***	0.01	1.47	−0.45	0.03	6.55 *	0.61 *
Liberal		0.01	0.27	−0.12	0.28	24.47 ***	−0.11	0.58 ***	0.01	0.76	−0.20	0.01	1.37	0.25
Ireland	IE	0.04	2.19	0.54	0.19	7.01 **	−0.05	0.44 ***	0.02	0.63	0.32	0.03	1.14	−0.40
UK	UK	0.05	4.07 *	−0.53 *	0.35	20.10 ***	−0.03	0.69 ***	0.04	3.95	−0.55	0.06	8.17 **	0.67 **

Notes: *** *p* < 0.001, ** *p* < 0.01, * *p* < 0.05. Source: Own elaboration from the data of the 6th EWCS 2016. © European Foundation for the Improvement of Living and Working Conditions (Eurofound), 2018 [41].
